# On the Puerperal Fever, and an Explanation of the Mode of Treating It

**Published:** 1802-12-01

**Authors:** 

**Affiliations:** Harbourg, in the Electorate of Hanover


### *On the Puerperal Fever, and an Explanation of the Mode of treating it; by* Dr. Michaelis, *of Harbourg, in the Electorate of Hanover.*

[ Continued from pp. 433—443 of our laſt. ]

I Generally preſcribe in the following manner: ℞. *Radic Valerian, unc. j. infund. in aqua fervent. q. s. Colatur. unc. vj. adde Salis Tartar. acet. vin. q. s. saturat. extr. Valerian. aa. drach, ij. or iij. extr. opii aquos. gr. iv.* S. omni bihorio coch-lear. duo, or half a tea-cup full; ſometimes I order it to be taken every hour. This medicine I continue during the whole diſorder; only ſometimes I omit the opium, when I find it too ſtimulating or too aſtringent; ſometimes I increaſe the doſe accordingly as new ſymptoms or a more violent diarrhœa make their appearances. Beſides theſe remedies, I foment the breaſts with a decoction of camomile flowers and black henbane, toge-ther with warm milk, cauſing the child, at the ſame time, to be applied to the breaſt, which undoubtedly is the ſtrongeſt attractive of the milk towards the breaſts; or by other ſubſtitutes for ſucking, creating a ſtimulus to that effect. By theſe fo-mentations, the ſpaſms in the breaſts, which prevent the ſecre-tion of the milk, are removed, and the operation of warmth and ſucking induces the fluid towards its proper parts. If the lochiæ be ſtopped immediately, or at leaſt too ſoon, by which the diſeaſe has become worſe, having loſt an equivalent for the former nutrition of the child, and for a moderate ſecretion of the milk; moreover, if little or no mucus diſcharges, and if the ſpaſms have alſo affected thoſe parts, I then cauſe injections to be made into the uterus, every two hours, of a ſimilar decoction, mixed with linſeed.

In order to act upon the uterus itſelf, and partly upon the the breaſts, ſince both are cloſely connected, and the defici-ency of ſecretion in one part is often ſupplied by that of the other, I particularly inſiſt upon thoſe injections being made, when the genitals have ſuffered by a difficult birth or continued ſpaſms, in which caſe I add oil and opium. But the principal remedy, either for adjuſting any irregularity in the proceſs of ſecretion and for removing the ſpaſm, or for promoting the reſorption of the milky fluid evacuated into the abdomen, the breaſt, and other parts, conſiſts in repeated bliſtering, to which, conformably to my obſervations, I muſt aſcribe the greateſt effect in the ſucceſsful treatment of this dangerous malady; I apply bliſters as ſoon as any part is afflicted with pain, and bring them as near to the place affected as poſſible.

It

It is always advifable, for the purpofe of enfuring fuccefs, not to delay the application of this remedy, though the fymptoms fhould appear to indicate no danger.

The rubbing in of volatile camphorated liniment is not without its ufe, but I commonly found it infufficient, and confider it merely as a fecondary remedy. I apply the blifter of the fize of an octavo page, and let it remain till it begins to draw a puftule; then I prefcribe a frefh blifter to be put on a place, as near to the former as poffible, whenever I am induced to apprehend a return of pain, an increafe of fever, and of milky evacuation through improper parts, or lacteal metaftafes, from the renewed fwelling of the abdomen, the oppreffion of the cheft, &c. It is often neceffary to apply from fix to eight blifters, one after another; they always produce the moft fpeedy effect, and the patients themfelves will demand this relief, as foon as they become acquainted with it by experience, difagreeable as it is to feel fore half over the body.

This, in general, is the method I adopt for the cure of a difeafe which feldom has yielded to common remedies, but rarely refifted my mode of treatment. Some particular fymptoms, however, require fome alterations in the treatment; in general, when the belly is chiefly affected, a violent diarrhœa, by which frequently a yellowifh watery matter, mixed with flakes of milk, is evacuated, accompanies the difeafe. This diarrhœa is uncommonly debilitating; it promotes the deviation of the milk from the breafts, and is often excited or increafed by too bold purgatives. It indeed procures a momentary relief, when the belly is very much diftended, and the milky fubftance has been extravafated into the bowels, nor fhould it be entirely ftopped: but when the milky fubftance has been thrown into the abdomen, it gives no eafe whatever; on the contrary, the belly fwells ftill more while it lafts.

In order to moderate this diarrhœa, yet not to obftruct evacuation altogether, the phyfician muft exert himfelf to procure proper remedies. If, for inftance, ftrong dofes of opium be not adequate, or if it be attended with danger to give this medicine in ftrong dofes, and the diarrhœa has continued for fome time, recourfe muft be had, at the fame time, to mucilaginous remedies, in which capacity I ufually employ falop. A few cafes may occur, in which coftivenefs takes place, greatly afflicting the patients, efpecially when the milky matter has found its way into the bowels. In fuch inftances, a motion fhould be procured by fome gentle aperient or a mild clyfter; but care muft be taken, not to excite any great diarrhœa; and fhould it have arifen, as often will happen even after very gentle purgings, care muft be taken to treat it in the proper manner.

Should

Should a difficult delivery, attended with the bruiſing or laceration of the perineum have preceded, ſo that there may be reaſon to apprehend an inflammation, or ſhould that inflammation already have taken place, injections into the vagina and uterus of lenient and oily ingredients muſt immediately be adopted after the delivery, in order to obviate that danger, or to remove it if it already exiſts. I then apply fomentations of camomile flowers, henbane, linſeed, and opium to the belly, and frequently cauſe ſome volatile camphorated ointment to be rubbed in. Inwardly I give oil mixed with the other medicines, which manifeſtly prove efficacious. It ſeldom happens that a lying-in woman, in caſe of inflammation, either in the vagina or the uterus, eſcapes without an attack of the puerperal fever, which, under thoſe circumſtances, often brings on a quick death. I loſt a patient ſome years ago, who, probably, by a groſs blunder of the midwife, had ſuffered a laceration of the vagina towards the uterus. The child had been dead ſeveral days when I was called in, and the uterus was filled with putrid ichor; the back of the child was turned foremoſt, but the delivery, owing to the relaxed ſtate of the uterus, and the putrid condition of the child, was quickly effected. However, when all was nearly done, one foot, after I had ſeized the leg with a hook, ſtuck faſt, together with the hook, in a hole of the vagina. By this laceration, I touched the bowels and the great arteries of the pelvis. A violent inflammation of the bowels enſued, which I was fortunate enough to remove. Five days afterwards, all ſigns of inflammation ſubſided, under the moſt favourable circumſtances. The belly became leſs, and free from pain, the cold extremities got warm, milk had begun to appear in the breaſts, and there was reaſon to hope for a ſucceſsful iſſue. The mode of treatment had been throughout of the ſtimulating kind; but all at once, things again grew worſe, the belly again became painful and extended, the milk receded from the breaſts, and a lacteal diarrhœa ſhowed but too plainly the unfortunate turn which the diſorder had taken. The woman died with ſigns of mortification, eight days after her delivery. In another caſe of a ſimilar nature, which ſoon after occurred, I was fortunate enough to ſave the patient. A laceration of the vagina exiſted likewiſe, but the infant only died during its difficult birth; a violent inflammation aroſe in the uterus, and ſeveral attacks of lacteal metaſtaſes followed; but, after conſiderable time, the recovery was at laſt effected. The woman, however, has not been with child ſince.

If the breaſt particularly ſuffers, it will be neceſſary, beſides the application of the bliſters to the affected ſide, to promote expectoration by means of Kermes and ſyrup of ſenega, which, which, in thefe inftances, fmells like four milk, and is of a white colour. If the breath be four like milk, oppreffed and unequal, we may fafely form the conclufion that the lungs are affected, although no milk is thrown up by coughing; and very great attention is then required. I have, in thefe cafes, caufed warm fteam to be inhaled, but without benefit. In cafe of the throat and larynx being affected, blifters muft be applied, and a gargle of vinegar and honey be made ufe of. When towards the end of the diforder, ftrength did not return, and the fever continued, I have given bark with the greateft fuccefs. The limbs, fometimes, fwell all over, without its appearing that matter, or an abfcefs of milk, had any where collected. Under thefe circumftances, I have found dry difcutient herbs with camphire, and alfo the volatile ointment with camphire, not always effectual in reducing the fwelling, and the laft patient of the kind I had, died under my hands. Should I again meet with a fimilar cafe, I fhall apply a blifter to the fwelling. To judge from one fubject I faw lately, it appears, that fuch a fwelling of the legs may degenerate into obftinate and indolent hard tumours, and indurations of the tela cellulofa. The milky fubftance may gather in one place, and form into pus; then it is advifable to bring this tumour to maturity by fomentations, and to open it as foon as poffible.

It is objected by fome, that by opening this fore too early, the depofition of milk towards this part would be prevented, and conducted to other parts; but it is furely more fafe to endeavour to check the propenfity of Nature towards fuch anomalous motion, and to obviate it by inward remedies. It often requires a long time before abfceffes of milk are healed. It is not to be denied, that lacteal metaftafes upon the cellular texture of the breafts may likewife arife; but whether every accumulation upon the breaft be of this kind, I will not undertake to determine, fince the diagnofis is not always eafy. Perhaps fome fixed pain in one part or other of the belly may remain after the difeafe, and the belly be, in fome particular fpot, hard and extended: it is then to be expected, that the milk which has been caft into the abdomen, has occafioned concretions of the inteftines, and other vifcera. In a cafe of that defcription, I ordered arnica to be taken inwardly, and gave relief by the rubbing in of camphorated ointment. Yet it is not to be expected, that the complaint will thoroughly be cured and the body reftored to its former ftate. Thefe concretions are by no means unfrequent, as likewife thofe which are the effect of inflamed lungs, and which, at firft, may caufe fome fenfation in cafe of any ftrong motion, but by degrees become imperceptible. At the fame time no one will wonder that

 barrenbarrennefs fhould be the confequence. I have frequently, when the belly was fwelled, made ufe of warm fomentations, and thus fometimes afforded relief. But not to mention the difficulty of applying them when the belly is covered with veficatories, I have often noticed an unfavourable effect; in confequence of which I now omit them entirely, except in cafe of inflammation. They certainly appear to conduct the milk towards the belly, and often increafe the tenfion and pain.

As to the diet to be followed in this malady, it fhould be thin and eafy to digeft, but, at the fame time, nutritive. I allow my patients broth, milk and water, gruel, and fago; wine and coffee I forbid as being too ftimulant; the former, however, I fometimes admitted, when diluted with water. Meat and all folid food, I judge would not agree, as the power of digeftion is commonly deranged. If the patients fhould defire an acid drink, it may, without prejudice, be granted; at leaft, I never obferved any bad effect from it. Beer and fermented liquors, and flatulent eatables, ought to be carefully avoided. Such is my manner of proceeding in a diforder generally attended with much danger. I have feldom failed of fuccefs, whenever I was called foon enough, and under circumftances fufficiently favourable, when I had it in my power to fee the patients daily, and had not neglected the veficatories on account of fymptoms appearing at firft to be flight.

Dr. *Brandes*, a Phyfician of eminence, maintains, that the puerperal fever is a lacteal metaftafis connected with a typhus, from which opinion my own does not very materially differ, though he fuppofes a metaftafis without an afthenic fever; whereas I am convinced, that even with the flighteft lacteal metaftafis an afthenic fever is connected, which when preceded by fome degree of debility in confequence of a ftrong ftimulus from the milky fubftance accumulated in the blood, may have a fthenic appearance, but will certainly pafs over into an afthenic fever. Let this, however, be as it may, the treatment differs but little, and is only in the lighter cafes lefs ftimulant. Blifters are indubitably very proper in every inftance.

Case I. A woman, 19 years of age, who had been married three years, of a pale appearance, and who had frequently been afflicted with a cough, but had, in other refpects, a good ftrong conftitution, was, for the firft time, delivered of a healthy child, and feemed, during the firft days, perfectly well. About the ninth day after her lying-in, when her breafts were filled with milk, fhe was feized with fhivering fits, without any obvious caufe, which were followed by frequent fweats and chillinefs; yet the ftate of her health in general was tolerably good, good, nor had the milk ſuffered any diminution. Not having had any motion of her bowels during the laſt three days of the ſecond week, a ſurgeon ordered her a laxative, from infus. laxativ. Viennens. which was not remarkably ſtrong, nor was the whole of it taken; but ſhe began in conſequence of it to purge conſiderably, and had more than five ſtools a day; ſhe unfortunately however met about this time with ſome fright. Upon this the milk diminiſhed, a reiterated chillineſs and violent heat followed, which was ſucceeded by a ſtrong ſweat. I was then called in to ſee her. I found the patient low in perſpiration with a pretty quick and ſpaſmodic pulſe; the belly was rather extended, and ſomewhat painful in the vicinity of the uterus when touched. In the preceding night ſhe had had five thin ſtools, deſtitute of ſmell; had ſuffered much from heat and ſweat, and been in ſome degree delirious; her throat was a little inflamed, the breaſts were flaccid, and the milk not ſufficient to ſatisfy the child. The lochiae flowed, mixed with mucus, as might be expected, yet not to a very great degree. It was but too plain, that lacteal metaſtaſes towards different parts were to be apprehended, and that the diſeaſe upon the whole was a true Puerperal Fever. I preſcribed as follows, ℞. *Pulver. rad. valer. sylvest. unc. j. infund. aq. fervent. q. s. ebull. paulisper, stet in vas. claus. ad refrigerat. colat. c. expres. unc. v. add. sal. tart. pur. dr. j. acet. vin. q. s. ad saturat. extr. opii aquos. gr. iij. syrup. emulsiv. unc. j.* M. D. S. Every two hours, half a tea cup full to be taken. ℞. *Spir. sal. ammon. caustic. dr. ij. camphor. dr. j. ol. olivar. unc. j. M. f. liniment.* D. S. to be applied to the belly and throat by rubbing in, to which was added a gargle from figs and honey, as I could not readily believe that a lacteal metaſtaſis had been carried to the throat, but was inclined to think it a common catarrhal complaint. Theſe remedies were continued the following day with apparent benefit. The diarrhœa had ceaſed, the milk was augmented, the fever leſſened, and no ſigns of delirium were obſerved in the night. But on the day following, which was October 9, I found the fever again increaſed, and the oppreſſion of the breaſt more conſiderable. At the ſame time a cough had made its appearance, in which a white ſouriſh matter was thrown out, having the ſmell of ſour milk. The breath likewiſe of the patient and the ſweat were attended with the ſame ſort of ſmell. The throat was again become more painful, and the milk and lochiae were diminiſhed. I ordered a bliſter to be applied to the upper part of the cheſt, the breaſts to be fomented with a decoction of chamomile flowers and milk, a gargle of vinegar and chamomile tea, a ſtrong infuſion of valerian with the extract of the ſame, together with opium and Huxham's wine; and laſtly, as no ſtool

 followed, followed, a clyfter, which however produced no effect. After this mode of treatment, the milky expectoration, and the oppreffion of the breaft ceafed, and only a little pain was felt near the ftomach. The fever itfelf was more moderate, but as the bowels continued unmoved, and the patient complained of fullnefs and anxiety, I ordered the following prefcription: ℞. *Magnes. alb. dr. j. cremor. tartar. dr. ij. sacchar. alb. dr. j. pulver. sem foenicul. dr. semis.* M. D. S. every three hours two tea fpoons full to be taken; and fhould the anxiety increafe, fome powders compofed of valerian and extr. hyofciam. were to be given.

Oct. 10. The powders were continued, and a clyfter given, but without producing an opening. The patient found herfelf pretty well, had little fever, no pains in her cheft, no expectoration, and fomewhat more of milk in the breafts. The four tafte however ftill exifted, and the pulfe was as yet fpafmodic; but as the pain about the ftomach increafed towards the evening, a blifter was applied there.

Oct. 11. The patient was pretty well, but had not much milk. No motion of the bowels having taken place, and the belly being rather full and extended, I prefcribed, ℞. *Pulver. rad. valerian. sylvest. unc. j. infund. aq. fervent. &c. col. unc. vj. add. infus. laxativ. Vienn. unc. iij. extr. valer. frigide parat. dr. iij. liquor. antispasmod. lent. dr. j.* M. D. S. every two hours half a tea cup full to be taken.

Oct. 12. Towards morning feveral ftools had taken place, which contained cafeous milk; only about half the medicine had been taken; the belly was painful and much extended; fever and fweat increafed, and the milk in the breaft ftill more diminifhed. A blifter was now immediately put upon the left fide of the abdomen as being the moft painful, the other part of the belly was rubbed with volatile camphorated ointment, and the breafts were frequently fomented. Inwardly the following was given, ℞. *Infus. rad. valer. unc. vj. extr. opii aq. gr. iij. extr. valerian. frig. parat. dr. ij. spir. nitr. dulc. dr. j.* M. D. S. every hour a table fpoon full to be taken. Befides this the child was applied to the breafts, and the milk drawn by other means; the patient was ordered not to be kept too hot. The vehement heat not having yet fubfided, towards the evening I prefcribed alkali faturated with vinegar, together with fyrup. acetos. citri, and ordered a tea fpoon full to be taken of this every hour or oftener. The other medicine was referved till the pain or diarrhœa fhould increafe; the latter was fomewhat lefs, but ftill milky. Upon this the heat confiderably decreafed, but the pains and oppreffion in the belly continued. For this reafon, I applied a blifter to the right fide of the abdomen, late in the evening.

In

In the night preceding the 13th, much anxiety and difquietude were experienced; there had been fome ftools of the fame kind, and the patient complained again of an oppreffed cheft, but there was no milky expectoration. Some milk appeared in the breaft, efpecially towards the afternoon and evening. Sal tartari faturated with vinegar was added to the infufion of valerian, and taken every hour. Towards the evening the fever was leffened and the patient felt more comfortable. Still however the belly was extended, though apparently lefs painful; I fay, apparently, becaufe it could not be exactly afcertained, as it was already covered with three blifters. The ftools this day were of the former defcription. The medicine was continued as before; but in cafe of obftruction and anxiety during the night, fome dofes of the medicine of October 11, together with the infus. laxativ. Vienn. were ordered. The fmell proceeding from the mouth was ftill four, but the four fweat had very much fubfided.

The night preceding the 14th of October was paffed in a quiet fleep without great heat. In the morning a feculent yellow ftool mixed with a little milk followed; the belly was lefs painful and lefs extended, the milk plentifully in the breaft, and the pulfe almoft of a natural quicknefs, and not very fpafmodic. The heat was moderate, and the fweat entirely gone; there only remained a degree of anxiety, but without a cough. The medicine was taken every two hours, fomentations and drawings continued, and the patient ordered to be kept cool and frequently expofed to frefh air. Milk and water, or toaft and water with lemon-juice, was recommended for her drink. In the evening fhe found herfelf pretty well, without fever, with little pain in the abdomen, and more milk in the breafts; fhe had had no motion to ftool. The fuccefs was very evident, and I think myfelf juftified in afcribing it to the mode of treatment I had adopted, and efpecially to the liberal ufe of blifters; but favourable as fymptoms now appeared to be, there was yet little ground for reckoning on a continued progrefs of recovery, as will appear from the following.

Oct. 15. The night paffed away without heat, thirft, or anxiety; the pulfe in the morning was almoft natural, but rather quick, and fomewhat fpafmodic. Two ftools of a pretty regular confiftency had been evacuated; the belly was but little painful; the tafte ftill four and flimy, and a flight cough remained, but without adding to the anxiety; the lochiae reappeared. The milk in the breafts was not fo plentiful as before, but almoft enough to fatisfy the child. Towards the evening, the pulfe feemed to be more irritated and feverifh; otherwife the patient was well enough to quit the bed.

 Oct.

Oct. 16. The recovery feemed to proceed, the appetite was increafed, only the pulfe was unequal. As this was ftill more the cafe in the evening, and the pulfe feverifh and fpafmodic, one of Dover's powders was taken.

In the night of the 17th, a great degree of fever, which had been preceded by chillinefs, took place with much heat, anxiety, ftitches in the fide, and pains in the belly; the tafte had become flimy, and of a putrid fournefs. In the morning therefore, early, a blifter was clapped to the painful fide and the medicine continued. About noon the pulfe was ftill very feverifh, quick, tenfe and full, but the ftitches had fubfided. The belly on the other hand was more extended and difturbed by a great deal of rumbling. I prefcribed an infufion of valerian, with the extract of valerian, opium, and mixtura Riverii. In the evening I found the patient better, lefs feverifh, and the pain diminifhed. Notwithftanding the pulfe was more than 100, fhe had had fome ftools of a good quality. A quiet night followed, though the patient talked much in her fleep; and in the morning the pain in the fide had entirely difappeared, though the belly when touched was not free from pain, and ftill entertained a fenfation of heavinefs. The evacuation was brown, and rather loofe; the pulfe ftill feverifh and fpafmodic, but lefs fo than the day before. The milk increafed in the breafts; the appetite was good, and the tafte improved. In the evening very little heat remaining, the pulfe 80, and fmall, little inconvenience in the belly except the heavinefs, nor was it much extended.

The night preceding the 19th, was quiet and comfortable, and the patient had milk enough to fuckle the child; but fuddenly the pains in the fides returned more violently than before, fhifted from the fide to the belly, then to the breaft, the fhoulders, the back, then to the abdomen and the regio lumborum, with fenfation of coftivenefs, and as if the menftrua were coming on. Some blood mixed with mucus really paffed off, as the lochiae had entirely ceafed the day before; the evacuation by ftool was brown, and frequent eructation was experienced. About nine o'clock a chillnefs arofe, and it was not till then that the deteriorated ftate of the patient was communicated to me.

I found the patient pale, with cold extremities, and a fmall fpafmodic and very tenfe pulfe of a 120, which as the pain increafed, refembled the vibration of a mufical ftring. The belly was extended and all over painful; I immediately gave two powders, one after another, of valerian and extr. hyofciam. together with fome camomile tea. Thefe produced a vomiting, by which fome relief was afforded. I then applied a blifter to the the ſide, which was ſtill free from pain, and gave, as the ſpaſm did not immediately ſubſide, a doſe of Dover's powders. The ſpaſmodic motion of the pulſe then ſeemed to abate, and in a few minutes after, a fit of heat came on; the pain, which continued ſhifting about, decreaſed without entirely ceaſing; another vomiting enſued, by which a great deal of browniſh water, together with phlegm, was thrown up. About noon, the patient was overwhelmed with heat, yet able to ſuckle her child. She now complained of a pain in her ſtomach and the belly. I cauſed pieces of woollen cloth, dipped in camomile tea, to be laid upon thoſe parts, and a clyſter from camomile infuſion and oil to be given. The medicine was continued. Towards the evening, the moveable pain had ſomewhat ſubſided, but the ſtomach and ſide ſtill ſuffered, and the fever was ſtrong. As the patient found it impoſſible, on account of the pain, to turn about, the clyſter could not be applied. To obviate the violent pains about the ſtomach, which perhaps were a conſequence of the exertion of vomiting, a fomentation of camomile flowers, of the leaves of hyoſciamus, of linſeed, and of opium, was prepared. The following night was reſtleſs and interrupted by talking and raving, the pain in the ſide had in the mean time diſappeared, and the fever was leſs violent. There was much ſweat, but no evacuation by ſtool. The place about the ſtomach and the belly were, when touched, ſtill painful and extended; the latter was very heavy, ſo as to fall to the ſide to which the patient turned. There was a tolerable quantity of milk, but the lochiae were quite ſtopped. As the patient had no motion to ſtool, ſhe ſuffered much oppreſſion and anxiety; it was alſo to be apprehended, that milk had again been thrown to the bowels, in conſequence of which the medicine of opium was ſuſpended, and, in the mean time, an ounce infus. laxat. Vienn. with half a ſcruple extr. valer. was given. The taſte was of a putrid ſourneſs, as well as the breath, and the tongue quite white. In the afternoon, a thin browniſh evacuation took place, without any milk mixed with it. The fever was more moderate towards the evening, the pains leſſened, and the breaſts pretty well filled with milk; the bad taſte, however, ſtill continued, and the patient again brought up, by means of a little cough, ſome ſour milky matter. All parts of the body in the vicinity of the painful ſpots being already covered with bliſters, it ſeemed of no uſe to add to the number of them. The medicine was continued.

The night preceding the 21ſt was again reſtleſs, the fever violent, the pain in the belly, and more particularly about the ſtomach and the navel conſiderable, and that in the right ſide, the ſhoulder and the back, returned occaſionally. The taſte

 retained retained that putrid fournefs, which it had before: the tongue was very foul. The cheft was ftill very uneafy, and a milky expectoration now and then took place. Though the lochiae did not flow, the milk was not diminifhed. As no evacuation followed, I again prefcribed the former purge; and as the fmell was extremely putrid, I gave vitriolic acid with rafpberry juice by itfelf, and partly in water. The body being every where fore, it was out of my power to add a new blifter, however profitable it might have been, by abridging the progrefs of the diforder. About the evening, fome eafe was obtained, and a diminution of the pain in the belly, by a ftool. The fever was more moderate, though ftill of fufficient ftrength; and frequently an exhaufting fweat broke out. An infufion of valerian with faturated vegetable alkali, but without the addition of opium, was taken: To relieve the breaft, I tried the fteam of camomile tea; but this the patient could not endure.

In the following night little fleep and great reftlefsnefs, but not fo much fever. Three offenfive ftools had been difcharged. The belly ached a little in the vicinity of the ftomach, and towards the right fide; the ftitches in the right fide were gone, but re-appeared in the left, and the breath was fetched deeply. Tafte and the fmell from the mouth were putrid, the tongue more clean. The ufual remedies were continued; the left fide, which was ftill painful, rubbed with volatile camphorated ointment, and the ftomach fomented. The appetite was tolerable, the looks improved, and the milk almoft fufficient. In the evening, little heat, the pulfe quick and tremulous. The belly lefs painful and lefs tumid. Some ftools. The breath more pure, the tafte and tongue improved. Two drachms of fpirit vitrioli had been confumed, and to them I am inclined to afcribe a part of the effect produced. A quantity of phlegm, not very milky, was thrown up.

October 23d. In the courfe of the preceding evening till the noon of this day, feven ftools, not copious, but very offenfive, fome of which contained a little coagulated milk. Every evacuation was preceded by fome pain. The night had been uneafy, but fome reft was enjoyed about the morning; pulfe more foft and lefs quick, breaft more eafy, and the pain in the left fide gone; milk almoft fufficient for the nourifhment of a pretty ftout child. To counteract the loofenefs of the bowels, fome opium was again added, the fomentations were omitted, and fome blifter ointment frequently applied to the place about the ftomach, which had again healed. Till evening, four more ftools, containing fome indigefted food. The night following, more quiet, but a great deal of thirft; the belly obftructed. Towards noon, a pulfe of 80, free from all fymptoms of fpafm.

The

The tongue moift and clean, the breath foul. The belly being very tenfe, and no opening having taken place, the medicine was for a while fufpended. The pulfe irregular, though not quicker. The night preceding the 25th of October, quiet; no ftools; the belly tenfe and painful; fome eruptions and an appearance of purples upon the breaft; heat increafed, pulfe quicker. I prefcribed one ounce of a laxative mixture with extract of valerian, which produced little effect. Every thing affumed a more unfavourable appearance; the tafte was more foul. In the night following, fome evacuations took place, which gave no relief. The eruptions continued; the belly ftill painful, fo as to render fomentations and even bliftering neceffary; a prefcription of infus. valer. with potio Riverii was given, which by affording eafe, prevented the application of the blifter intended to be laid on. Tafte unclean, fmell putrid, and fome ftools.

October 27. The eruption began to difappear; the fever lefs; fome milky ftools. In order to prevent fuch frequent returns, which were chiefly to be attributed to weaknefs, that apparently required an inciting remedy, I tried the extract of the bark, of which I defired one dr. and a half to be diluted in fome ounces of peppermint water, after being mixed with the fame quantity of extr. valer. This was added to the former medicine, of which about three ounces remained, and the patient took every hour a table fpoon full of it; the heat after this did not increafe; the belly was lefs painful. I now ordered extract. cort. Peruv. to be taken by itfelf; but as fome degree of pain ftill remained in the left fide of the abdomen, efpecially when the patient lay on that fide, and as there was a ftrong tenefmus, I added oil of almonds and gum arabic, but no perceptible advantage was derived from it. The pain probably arofe from milk being poured into the abdomen, and being conftipated there: this feemed to be the cafe, on account of the heavinefs of the belly, befides which there were no particular indications.

From the firft of November, I fufpended the ufe of medicine; the milk was pretty abundant. The fixed pains ftill remaining, I prefcribed, on the 6th of November, an infus. fummitat. arnicae, to promote the reforption of the condenfed milky matter: this remedy, however, was obliged to be interrupted by reafon of a catarrh, which was the confequence of an epidemic, prevalent at that time. The patient, after this, continued to fuckle her child. She fuffered ftill from pain in the belly for a confiderable time, which now has difappeared, though it has left this part rather tumid. A new pregnancy has not yet taken place.

*Experiments*

